# Patterns of postmeal insulin secretion in individuals with sulfonylurea-treated *KCNJ11* neonatal diabetes show predominance of non-K_ATP_-channel pathways

**DOI:** 10.1136/bmjdrc-2019-000721

**Published:** 2019-12-18

**Authors:** Pamela Bowman, Timothy J McDonald, Bridget A Knight, Sarah E Flanagan, Maria Leveridge, Steve R Spaull, Beverley M Shields, Suzanne Hammersley, Maggie H Shepherd, Robert C Andrews, Kashyap A Patel, Andrew T Hattersley

**Affiliations:** 1 Institute of Biomedical and Clinical Science, University of Exeter Medical School, Exeter, Devon, UK; 2 Exeter NIHR Clinical Research Facility, Exeter, Devon, UK; 3 Royal Devon and Exeter NHS Foundation Trust, Exeter, Devon, UK

**Keywords:** permanent Neonatal Diabetes, SU (Sulfonylurea), insulin secretion, physiology

## Abstract

**Objective:**

Insulin secretion in sulfonylurea-treated *KCNJ11* permanent neonatal diabetes mellitus (PNDM) is thought to be mediated predominantly through amplifying non-K_ATP_-channel pathways such as incretins. Affected individuals report symptoms of postprandial hypoglycemia after eating protein/fat-rich foods. We aimed to assess the physiological response to carbohydrate and protein/fat in people with sulfonylurea-treated *KCNJ11* PNDM.

**Research design and methods:**

5 adults with sulfonylurea-treated *KCNJ11* PNDM and five age, sex and body mass index-matched controls without diabetes had a high-carbohydrate and high-protein/fat meal on two separate mornings. Insulin(i) and glucose(g) were measured at baseline then regularly over 4 hours after the meal. Total area under the curve (tAUC) for insulin and glucose was calculated over 4 hours and compared between meals in controls and *KCNJ11* cases.

**Results:**

In controls, glucose values after carbohydrate and protein/fat were similar (median glucose tAUC_0-4h_21.4 vs 19.7 mmol/L, p=0.08). In *KCNJ11* cases glucose levels were higher after carbohydrate than after protein/fat (median glucose tAUC_0-4h_58.1 vs 31.3 mmol/L, p=0.04). These different glycemic responses reflected different patterns of insulin secretion: in controls, insulin secretion was greatly increased after carbohydrate versus protein/fat (median insulin tAUC_0-4h_727 vs 335 pmol/L, p=0.04), but in *KCNJ11* cases insulin secretion was similar after carbohydrate and protein/fat (median insulin tAUC_0-4h_327 vs 378 pmol/L, p=0.50).

**Conclusions:**

Individuals with sulfonylurea-treated *KCNJ11* PNDM produce similar levels of insulin in response to both carbohydrate and protein/fat meals despite carbohydrate resulting in much higher glucose levels and protein/fat resulting in relatively low glucose levels. This suggests in an inability to modulate insulin secretion in response to glucose levels, consistent with a dependence on non-K_ATP_ pathways for insulin secretion.

**Trial registration number:**

NCT02921906.

**Significance of this study**

**What is already known about this subject?**

- Individuals with *KCNJ11* PNDM can be successfully treated with high-dose sulfonylureas which maintain excellent glycemic control without causing severe hypoglycemia.
- Some individuals with sulfonylurea-treated *KCNJ11* PNDM report mild-moderate postprandial hypoglycemia, particularly after protein/fat-rich meals.

**What are the new findings?**

- Individuals with sulfonylurea-treated *KCNJ11* permanent neonatal diabetes mellitus (PNDM) exhibit higher glucose values in response to a carbohydrate meal than to a protein meal, and for both meals glucose levels are higher than controls without diabetes, who exhibit much more tightly regulated glucose levels regardless of meal type.
- The more tightly regulated glucose levels in controls results from much higher insulin secretion in response to carbohydrate than to protein, whereas cases with sulfonylurea-treated *KCNJ11* PNDM have similar insulin levels in response to both meal types.
- The similar insulin secretion with the different meals in individuals with sulfonylurea-treated *KCNJ11* PNDM suggests a relative inability to modulate insulin secretion in response to both higher (carbohydrate) and lower (protein/fat) glucose levels, consistent with a dependence on non-K_ATP_ pathways for insulin secretion.

**Significance of this study**

**How might these results change the focus of research or clinical practice?**

- Individuals with sulfonylurea-treated *KCNJ11* PNDM should avoid meals lacking carbohydrate or missing meals to mitgate the risk of postprandial hypolycemia.
- Future research in individuals with sulfonylurea-treated *KCNJ11* PNDM will focus on assessing glucagon secretion at low levels of glucose, the counter-regulatory response to hypoglycemia, and the effects of different doses and types of sulfonylurea on the physiological response to different meals.

## Introduction

Activating *KCNJ11* mutations are the most common cause of permanent neonatal diabetes mellitus (PNDM), diagnosed in the first 6 months of life.^1^ *KCNJ11* encodes Kir6.2, the pore-forming subunit of the ATP-sensitive potassium (K_ATP_) channel that, in the beta cell, closes in response to metabolically generated ATP, causing beta cell depolarization and insulin secretion. The SUR1 subunit, encoded by the *ABCC8* gene, further regulates K_ATP_ channel activity by opening the channel in response to Mg-ADP, preventing insulin release.^2^ *KCNJ11* mutations impair the ATP sensitivity of pancreatic K_ATP_ channels rendering them unresponsive to rising blood glucose and the beta cell remains hyperpolarized,^3^ resulting in absolute insulin deficiency. Affected individuals required treatment with replacement doses of insulin until it was shown that sulfonylureas (SU) could bind and close pancreatic K_ATP_ channels resulting in beta cell depolarization and endogenous insulin secretion.^4^ This allowed 90% of individuals with *KCNJ11* PNDM to stop insulin injections completely gaining excellent metabolic control which is maintained long term.^4 5^

Severe hypoglycemia is rarely observed in SU-treated *KCNJ11* PNDM.^5 6^ This is remarkable given that the doses of SU used in affected individuals are around 5–10 times those used to treat type 2 diabetes (T2D) and indicates that SU-stimulated K_ATP_ channel activity is regulated, at least in part, in the presence of *KCNJ11* mutations. However, as these mutations prevent regulation by ATP, it has been suggested that non-K_ATP_-channel-mediated amplifying pathways of insulin secretion (eg, incretin hormones) predominate over the classical ATP pathway.^4^ This is supported by the very low levels of insulin secretion observed after intravenous glucose in comparison to oral glucose or meals, in individuals with SU-treated *KCNJ11* PNDM.^4^ The role of SUs is assumed to be largely permissive in allowing the beta cell to respond to non-K_ATP_-channel-mediated amplifying pathways, as opposed to directly stimulatory.^4^

Anecdotal reports from patients with *KCNJ11* PNDM have suggested mild to moderate hypoglycemia occurs after meals rich in protein/fat and lacking carbohydrate^7^ or meals smaller than usual in size.^6^ This may reflect an inability to moderate food-stimulated insulin secretion in the context of falling glucose after a low-carbohydrate meal; both GLP-1 and nutrient stimulation of the beta cell may play a role, as fatty acids and amino acids can drive insulin secretion through ATP-independent as well as ATP-dependent pathways.^8–11^ Similarly, K_ATP_ channels on pancreatic alpha cells^12^ and glucose-sensing neurons in the brain ventromedial hypothalamus^13^ are thought to play a role in counter-regulatory responses to hypoglycemia via ATP-dependent and independent mechanisms.^14 15^ SU inhibition and protein can both stimulate alpha cell depolarization and glucagon secretion in vitro.^16 17^ However, counter-regulatory processes are complex and remain incompletely understood.

Despite its clinical importance in relation to dietary advice and hypoglycemia risk, no studies have investigated the impact of protein or other food types on insulin or glucagon secretion in SU-treated *KCNJ11* PNDM. We therefore aimed to assess the insulin, glucose and glucagon response to carbohydrate and protein/fat in people with *KCNJ11* PNDM, and to compare this with the physiological response to the same food types in individuals without diabetes.

## Research design and methods

### Participants

Five adults >18 years of age with SU-treated *KCNJ11* PNDM and five controls without diabetes matched for age, sex and body mass index participated in the study. Clinical characteristics of the study participants are shown in table 1. All measured characteristics were similar between the groups except fasting glucose which was higher in cases.

**Table 1:** Clinical characteristics of study participants. All continuous numerical data are presented as median (range) unless otherwise stated. In one patient who was taking gliclazide and not glibenclamide, dose was converted to glibenclamide equivalent using % maximum dose according to British National Formulary

Clinical feature	*KCNJ11* cases	Non-diabetic controls	P value
Age (years)	39.1 (24.4–41.0)	39.6 (24.2–41.8)	0.60
Sex, male (%)	1 (20)	1 (20)	1.00
BMI	22.9 (22.4–26.8)	24.6 (23.9–25.6)	0.60
Fasting glucose (mmol/mol)	10.1 (8.6–11.9)	5.3 (4.8–5.5)	0.009
*KCNJ11* mutation	4 R201H, 1 R201C	N/A	N/A
SU dose (mg/kg/day glibenclamide)	0.28 (0.07–1.21)	N/A	N/A
HbA1c (%)	6.9 (6.5–7.9)	N/A	N/A
HbA1c (mmol/mol)	52 (48–63)	N/A	N/A

BMI, body mass index; N/A, not applicable; SU, sulfonylurea.

### Experimental procedure

Participants attended the Exeter NIHR Clinical Research Facility and were given a high-carbohydrate breakfast (77 g carbohydrate, 9 g protein, 1 g fat, 371 calories) consisting of orange juice and white toast with jam, and a high-protein/fat breakfast (46 g protein, 18 g fat, 6 g carbohydrate, 369 calories) consisting of ham and cheese, on two separate mornings in a random order. Participants with *KCNJ11* PNDM took their usual prescribed dose of SU (five on glibenclamide, one on gliclazide) with each breakfast. These individuals also took part in a third visit during which they did not have any breakfast but took their SU tablet as usual that morning.

Before each visit the participants fasted overnight for a minimum of 10 hours. Prior to breakfast an intravenous cannula was inserted and two baseline blood samples (−5, 0 min) were taken for measurement of insulin, glucose, and glucagon. Breakfast was provided, on visits 1 and 2, and participants were given 15 min to eat. Participants were also given a glass of water and 1000 mg paracetamol with the meal as a non-invasive measure of gastric emptying.^11^ Any food remaining after 15 min was removed and weighed. One control participant and one case did not finish the protein meal within 15 min (they ate 90% and 75% of the breakfast, respectively). All participants ate the full carbohydrate meal. Blood samples were taken at regular intervals after breakfast (every 15 min for the first hour then every half hour for the last 3 hours); they were immediately centrifuged and frozen at −80°C for later measurement of glucose, insulin, glucagon, and paracetamol levels. Participants were also screened at each time point for autonomic and neuroglycopenic symptoms of hypoglycemia using standard questions scored from 1 to 7 on a Likert scale as previously described.^12 13^ For the individuals with *KCNJ11* PNDM, the third visit involved the same procedure but without any food.

### Biochemical analysis

All biochemical analyses were performed in the Royal Devon and Exeter NHS Foundation Trust Clinical Laboratory. Serum glucose and paracetamol were analyzed on the 702 module of the Cobas 8000 analyzer, serum insulin was analyzed on the 602 module of the Cobas 8000 analyzer, and serum glucagon was analyzed on the Dynex DS2 automated ELISA platform.

### Statistical analyses

Data were analyzed in Stata V.14.2 using non-parametric statistical methods; Wilcoxon signed-rank test for paired continuous data and Mann-Whitney test for unpaired continuous data (independent samples). For categorical data, Fisher’s exact test was used for between-group comparisons. Total area under the curve over 4 hours (tAUC_0-4h_) and incremental area under the curve (iAUC_0-4h_) for insulin, glucagon and glucose were calculated using the trapezoidal rule. Values are reported as median (range) throughout unless stated otherwise.

### Glucose levels and hypoglycemia

Glucose trends after meals and responses to hypoglycemia questionnaires were described and glucose tAUC_0-4h_ and iAUC_0-4h_ were compared between meals in controls and *KCNJ11* cases.

### Postmeal insulin secretion

Insulin tAUC_0-4h_ and iAUC_0-4h_ were compared between meals in controls and *KCNJ11* cases. As baseline glucose values were different between cases and controls (table 1, online supplementary figure S1), insulin was also adjusted for glucose by calculating the ratios of total AUC for insulin and glucose (insulin tAUC/glucose tAUC)_0-4h_.^18^

10.1136/bmjdrc-2019-000721.supp1 Supplementary data

### Postmeal glucagon secretion

Glucagon tAUC_0-4h_ and iAUC_0-4h_ were compared between different meals in controls and *KCNJ11* cases.

### Gastric emptying

Maximum serum paracetamol concentration (p_max_) and time to maximum concentration (t_max_) were used to calculate the emptying index (t_max_ / p_max_), which was compared between different meals to check for differences in rates of gastric emptying as previously described.^19^ Paracetamol tAUC_0-4h_ was also compared between controls and *KCNJ11* cases and between meals.

### Effect of SU without food

To examine the effect of SU alone, the glucose, insulin and glucagon analyses were repeated in *KCNJ11* cases after no food and compared with the responses to the carbohydrate and protein/fat meals.

### Data cleaning

In the process of data analysis, for glucagon values <1.5 (limit of detection of the assay) a value of 1.4 was used. For paracetamol values <1.2 (limit of detection of the assay) a value of 1.1 was used. The baseline values for each analysis were an average of the minus-5-minute (−5 min) and 0-minute (0 min) values; if the −5 min or 0 min baseline value was missing, the single remaining baseline value was used. Where a value at a single time point was missing, an average of the values at the time points either side was imputed. On the three occasions where a sample was delayed due to the participant requiring recannulation, the result was allocated to the closest scheduled time point.

## Results

### Different glucose levels after protein/fat and carbohydrate in *KCNJ11* cases versus controls

**Figure 1:**
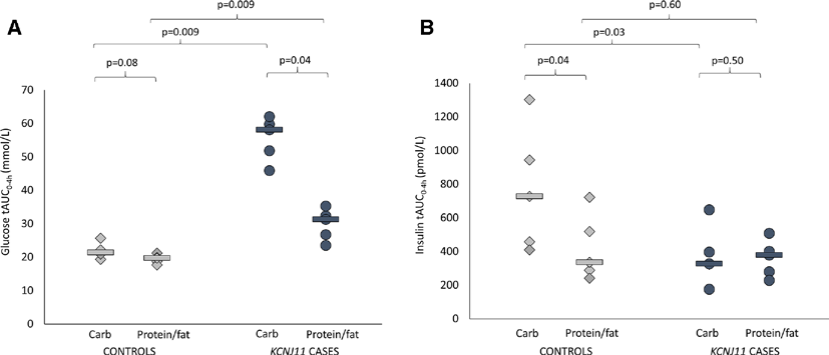
(A) Glucose total area under the curve (AUC) over 4 hours. Controls are shown in light gray (diamonds are individuals and lines are group medians). *KCNJ11* cases are shown in dark gray (circles are individuals and lines are group medians). (B) Insulin total AUC over 4 hours. Controls are shown in light gray (diamonds are individuals and lines are group medians). *KCNJ11* cases are shown in dark gray (circles are individuals and lines are group medians).

**Figure 2:**
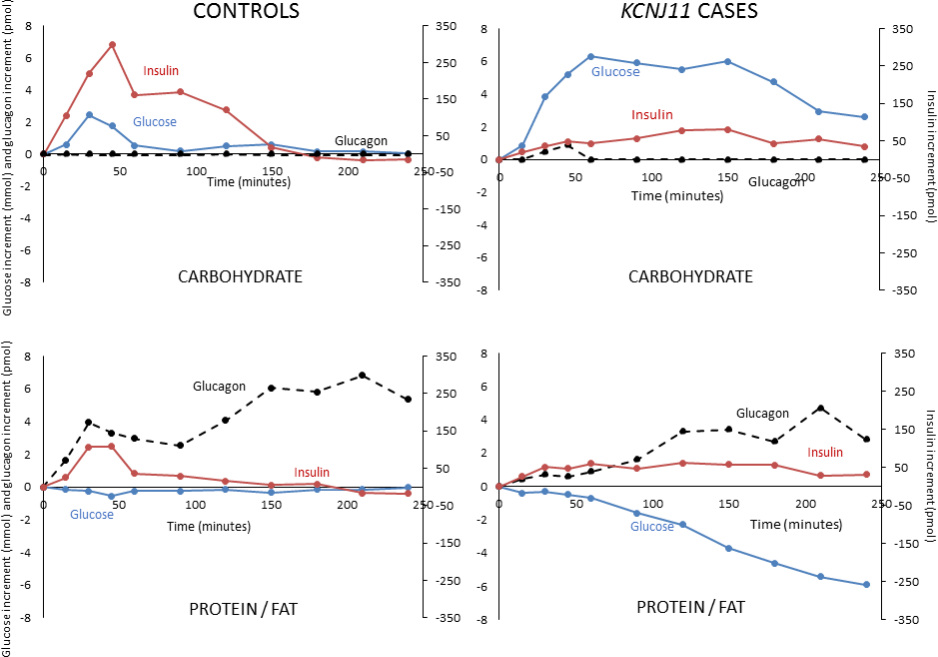
Incremental glucose (blue solid line), insulin (red solid line) and glucagon (black broken line) in controls without diabetes and sulfonylurea-treated *KCNJ11* cases with carbohydrate (upper panel) and protein/fat (lower panel) meals. Values shown are medians.

Glucose levels were higher in cases versus controls after both carbohydrate (glucose tAUC 58.1 (45.9–62.0) mmol/L vs 21.4 (19.3–25.7) mmol/L, p=0.009) and protein/fat (glucose tAUC 31.3 (23.5–35.3) mmol/L vs 19.7 (17.7–21.2) mmol/L, p=0.009) (figure 1A). In controls, glucose values were tightly regulated after the two meals (figures 1A and 2 and online supplementary figure S1) (glucose tAUC after carbohydrate 21.4 (19.3–25.7) mmol/L and after protein/fat 19.7 (17.7–21.2) mmol/L, p=0.08). In *KCNJ11* cases glucose levels were much higher after carbohydrate (glucose tAUC after carbohydrate 58.1 (45.9–62.0) mmol/L than after protein/fat 31.3 (23.5–35.3) mmol/L, p=0.04) (figures 1A and 2 and online supplementary figure S1). Similar trends were seen using iAUC (online supplementary table 1).

10.1136/bmjdrc-2019-000721.supp2 Supplementary data

In cases, glucose increased 6.3 mmol/L from baseline in the first hour after carbohydrate and remained 2.6 mmol/L above baseline at 4 hours. In contrast, after protein/fat, glucose fell reaching median 5.9 mmol/L below baseline at 4 hours (figure 2 and online supplementary figure S1). Despite falling glucose after protein/fat, no cases became hypoglycemic in the 4-hour study period: the lowest glucose value recorded was 4.4 mmol/L. Consistent with these glucose profiles, all cases and controls had the lowest possible scores at all time points when screened for symptoms of hypoglycemia, indicating an absence of subjective symptoms. There were also no objective symptoms of hypoglycemia during observation of participants by the study nurse and doctor.

### Different glycemic responses to meals were explained by different patterns of insulin secretion in *KCNJ11* cases versus controls

Insulin secretion was higher in controls versus cases after the carbohydrate meal (insulin tAUC_0-4h_ 727 (409–1302) vs tAUC_0-4h_ 327 (175–647) pmol/L, p=0.03) but not after the protein/fat meal (insulin tAUC_0-4h_ 335 (241–722) vs 378 (228–508) pmol/L, p=0.60) (figure 1B). In controls insulin secretion was greatly increased after the carbohydrate meal compared with the protein/fat meal (insulin tAUC_0-4h_ 727 (409–1302) vs 335 (241–722) pmol/L, p=0.04), but in the *KCNJ11* cases insulin secretion was similar with the two meals (insulin tAUC_0-4h_ 327 (175–647) after carbohydrate and 378 (228–508) pmol/L after protein/fat, p=0.50) (figures 1B and 2 and online supplementary figure S1).

The same pattern was observed using iAUC (online supplementary table 1) and when insulin secretion was adjusted for glucose (insulin tAUC/glucose tAUC)_0-0.4h_ in controls after carbohydrate versus protein/fat, 33.9 (19.6–50.8) vs 18.9 (11.4–36.2), p=0.04, and in *KCNJ11* cases 6.2 (2.9–14.1) vs 11.7 (9.7–14.4), p=0.08 (online supplementary figure S2). These results are consistent with *KCNJ11* cases not being glucose responsive, in contrast to controls without diabetes.

10.1136/bmjdrc-2019-000721.supp3 Supplementary data

### Glucagon secretion is increased in response to protein/fat compared with carbohydrate in both cases and controls

**Figure 3:**
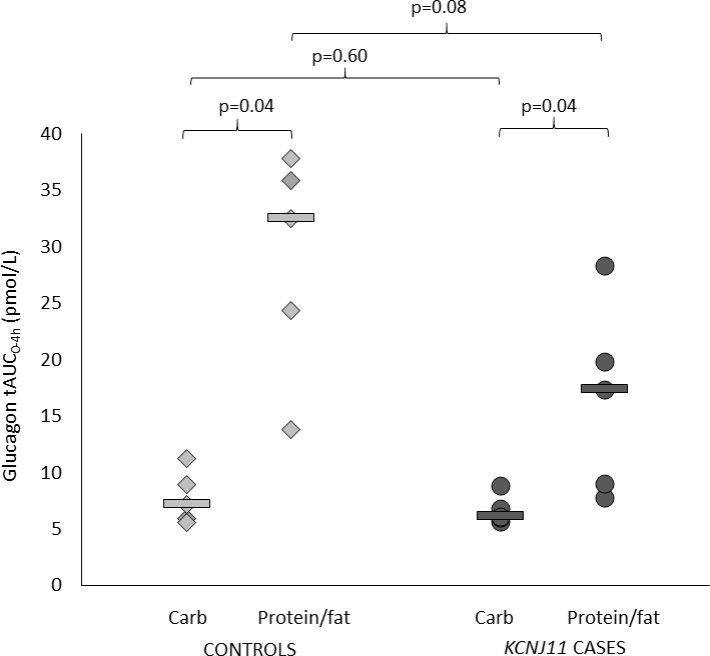
Glucagon total area under the curve (AUC) over 4 hours. Controls are shown in light gray (diamonds are individuals and lines are group medians). *KCNJ11* cases are shown in dark gray (circles are individuals and lines are group medians).

Both controls and cases had higher glucagon secretion after protein/fat than carbohydrate (glucagon tAUC_0-4h_ in controls 32.5 (13.8–37.9) vs 7.2 (5.6–11.2) pmol/L, p=0.04, and in cases 17.4 (7.8–28.3) vs 6.1 (5.7–8.9) pmol/L, p=0.04) (figures 2 and 3 and online supplementary figure S1). This is consistent with an alpha cell response to amino acids and/or fatty acids in both groups.

### Paracetamol profiles indicate similar rates of gastric emptying

Paracetamol concentration-time curves are shownin online supplementary figure S3. Emptying index (t_max_/p_max_) and tAUC were similar between controls versus cases (t_max_/p_max_ for carbohydrate 0.03 vs 0.05, p=0.46, and for protein/fat 0.04 vs 0.03, p=0.60, paracetamol tAUC_0-4h_ for carbohydrate 30.5 (21.9–55.5) vs 28.5 (18.5–44.4) mg/L, p=0.75, and for protein/fat 28.4 (24.4–46.7) vs 36.5 (13.6–40.4) mg/L, p=0.92). Emptying index and tAUC were also similar between meals in controls (emptying index p=0.89, tAUC_0-4h_, p=0.45) and in cases (emptying index p=0.50, tAUC_0-4h_, p=0.92).

10.1136/bmjdrc-2019-000721.supp4 Supplementary data

### Effects of SU independent of food in *KCNJ11* cases

Glucose fell to a similar extent after both SU only (no food) and protein/fat (online supplementary figures S4 and S5) and there was no difference in overall glucose levels (glucose tAUC_0-4h_ with no food 34.6 (28.7–42.8) mmol/L and after protein/fat 31.3 (23.5–35.3) mmol/L, p=0.22). In contrast, glucose levels were lower after SU only versus carbohydrate (glucose tAUC_0-4h_ with no food 34.6 (28.7–42.8) vs 58.1 (45.9–62.0) mmol/L, p=0.04). The same trends were seen using glucose iAUC (online supplementary table 1).

10.1136/bmjdrc-2019-000721.supp5 Supplementary data

10.1136/bmjdrc-2019-000721.supp6 Supplementary data

Insulin secretion in the 4 hours after a meal was higher after both carbohydrate versus SU only (insulin tAUC_0-4h_ 327 (175–647) vs 174 (129–280) pmol/L, p=0.04, and insulin iAUC 205 (104–480) vs 39 (13–101) pmol/L, p=0.04). There was also a trend towards higher insulin secretion after protein/fat versus SU only (insulin tAUC_0-4h_ 378 (228–508) vs 174 (129–280) pmol/L, p=0.08, and insulin iAUC 183 (109–316) vs 39 (13–101) pmol/L, p=0.04) (online supplementary figures S4 and S5). This supports a key role for food in triggering insulin release in the context of SU-treated *KCNJ11* PNDM.

Glucagon secretion was similar in the absence of food versus carbohydrate (glucagon tAUC_0-4h_ 6.0 (5.6–18.7) vs 6.1 (5.7–8.9) pmol/L, p=0.69, and glucagon iAUC 0.2 (−0.2 to 1.9) vs 0.5 (0.1–1.2) pmol/L, p=0.50) despite very different glucose levels. In contrast, there was a trend towards lower glucagon secretion in the absence of food versus protein/fat (glucagon tAUC_0-4h_ 6.0 (5.6–18.7) vs 17.4 (7.8–28.3) pmol/L, p=0.22, and glucagon iAUC_0-4h_ 0.20 (–0.2 to 1.9) vs 11.8 (2.2–16.7) pmol/L, p=0.04).

The rate of gastric emptying did not change with SU only in comparison to the two meals (paracetamol tAUC_0-4h_ with no food 34.6 (20.0–43.4) mg/L and with carbohydrate 28.5 (18.5–44.4) mg/L, p=0.89, paracetamol tAUC_0-4h_ with no food 34.6 (20.0–43.4) mg/L and with protein/fat 36.5 (13.6–40.4) mg/L, p=0.69).

## Discussion/conclusions

We have shown clear differences in glucose levels and insulin secretion after carbohydrate and protein/fat meals between controls without diabetes and individuals with SU-treated *KCNJ11* PNDM.

While controls show tightly regulated glucose levels after meals, individuals with *KCNJ11* PNDM have lower glucose after protein/fat versus carbohydrate. These different glucose profiles have clinical implications in terms of dietary advice offered to patients with SU-treated *KCNJ11* PNDM. In this specific type of diabetes, patients should avoid meals or diets completely lacking carbohydrate since blood glucose is likely to fall postprandially in the context of a protein/fat meal. Furthermore, they should avoid missing meals after taking SU, as we also observed a fall in glucose with SU in the absence of food.

In addition to the practical implications, our data may provide a mechanistic explanation as to why patients with SU-treated *KCNJ11* PNDM report hypoglycemia after protein/fat-rich meals. Insulin secretion after protein/fat and carbohydrate is similar in affected individuals despite very different glucose profiles. This supports insensitivity to glucose and lack of moderation of incretin and amino acid/fatty acid-stimulated insulin secretion after a protein/fat meal. Consistent with this is human and rodent data from previous studies demonstrating non-K_ATP_-driven insulin secretion in the presence of normal or low glucose. Humans with congenital hyperinsulinism caused by recessively inherited inactivating (loss of function) K_ATP_ channel mutations have insensitivity to leucine, which acts through K_ATP_ pathways, but sensitivity to glutamine, which acts independently of ATP to drive insulin secretion and hypoglycemia after protein-rich meals.^20^ Furthermore, SUR1 knockout mice are euglycemic but show amino acid-stimulated insulin secretion which is particularly sensitive to glutamine.^21^

The pattern of the insulin response to the different meals in SU-treated *KCNJ11* PNDM contrasts with the pattern in controls without diabetes where carbohydrate, acting through the classical ATP pathway, elicits a far greater insulin response than protein/fat and is quickly ‘switched off’ in response to normalization of blood glucose. It also contrasts with the response in individuals with type 1 diabetes on intensive insulin therapy, who show rises in postprandial glucose 3–5 hours after a high-protein/fat meal and in whom protein is protective against hypoglycemia.^22^

Our results are consistent with the previously hypothesized mechanism of insulin secretion in patients with SU-treated *KCNJ11* PNDM. Pearson *et al* reported a large reduction in insulin secretion following intravenous glucose versus oral glucose supporting predominance of non-K_ATP_-mediated amplifying pathways over the classical ATP pathway in these patients.^4^ The small amount of insulin secretion and fall in glucose we observed in the absence of food contrasts with the idea of a purely permissive action of SUs on the beta cell,^4^ although our experimental design differed from previous physiological studies limiting direct comparison. The very high doses of SU used to treat *KCNJ11* PNDM do not result in severe hypoglycemia highlighting the possibility of a different pharmacological mechanism to the direct effects on the K_ATP_ channel seen in T2D. Further research is needed to improve understanding of the mechanism of SU action in *KCNJ11* PNDM.

In our study, glucagon secretion was higher after protein/fat versus carbohydrate in both cases with *KCNJ11* PNDM and controls without diabetes, consistent with the previously described stimulatory effect of amino acids on alpha cells.^17^ We did not test the glucose responsiveness of alpha cells in this study as all individuals remained euglycemic throughout. However, rodent models suggest defective glucagon secretion may occur in the presence of K_ATP_ channel mutations. Specifically, SUR1 knockout mice show an alpha cell secretory defect at low levels of glucose,^23^ *KCNJ11* knockout mice exhibit defective glucagon secretion due to an impaired brain response to hypoglycemia,^24^ and intracerebroventricular perfusion of K_ATP_ channels with SU in conscious rats reduces glucagon responses to hypoglycemia.^25^ In humans, the research and anecdotal clinical evidence to date suggests that these patients are protected from severe hypoglycemia, but the mechanisms of this remain unknown and future research will investigate in detail the counter-regulatory response to hypoglycemia in people with *KCNJ11* mutations at the level of the alpha cell and the brain as previously described in the context of glucokinase mutations.^26^

Our study has important strengths. To our knowledge, it is the first study to assess the impact of different food types on glucose levels and insulin and glucagon secretion in people with *KCNJ11* PNDM, and to compare this with data from controls without diabetes. Previous research has been limited to assessment of insulin secretion following oral and intravenous glucose tolerance tests in small groups of affected individuals without a control group for comparison.^4 5^

Our study has some limitations. First, the numbers of cases and controls are small and only two mutations in the *KCNJ11* gene (R201H and R201C) were studied, reflecting the rarity of the disease. However, these are the most common mutations and both impact ATP binding. Second, as the individuals in our study did not have glucose levels in the hypoglycemic range, we were unable to assess the alpha cell response in the presence of low glucose as discussed above. Finally, the study was only done in adults, which may limit the generalizability of the findings, particularly for patients in the pediatric age range. Additional studies in children with SU-treated *KCNJ11* PNDM will be required to investigate the beta and alpha cell responses to carbohydrate and protein/fat and to establish whether these responses differ from those observed in adults.

In conclusion, we have shown that individuals with SU-treated *KCNJ11* PNDM produce similar levels of insulin in response to both carbohydrate and protein/fat meals despite carbohydrate meals resulting in much higher glucose levels and protein/fat meals being characterized by relatively low glucose levels. This suggests an apparent inability to modulate insulin secretion in response to both higher (carbohydrate) and lower (protein/fat) glucose levels, which is consistent with a dependence on non-K_ATP_-channel pathways for insulin secretion. Our findings may provide a mechanistic explanation for the postprandial hypoglycemia reported by patients with *KCNJ11* PNDM. Furthermore, glucose levels can fall with SUs in the absence of food. We would therefore recommend that affected individuals avoid missed meals or meals lacking carbohydrate while on SU treatment. Finally, our data highlight the utility of *KCNJ11* PNDM as a model for studying non-K_ATP_-mediated pathways of insulin secretion and demonstrate the predominance of the classical ATP pathway in the non-diabetic state.

10.1136/bmjdrc-2019-000721.supp7 Supplementary data

## References

[1] De Franco E, Flanagan SE, Houghton JAL, et al The effect of early, comprehensive genomic testing on clinical care in neonatal diabetes: an international cohort study. Lancet 2015;386:957–63. 10.1016/S0140-6736(15)60098-8 26231457 PMC4772451

[2] Babenko AP, Polak M, Cavé H, et al Activating mutations in the ABCC8 gene in neonatal diabetes mellitus. N Engl J Med 2006;355:456–66. 10.1056/NEJMoa055068 16885549

[3] Gloyn AL, Pearson ER, Antcliff JF, et al Activating mutations in the gene encoding the ATP-sensitive potassium-channel subunit Kir6.2 and permanent neonatal diabetes. N Engl J Med 2004;350:1838–49. 10.1056/NEJMoa032922 15115830

[4] Pearson ER, Flechtner I, Njølstad PR, et al Switching from insulin to oral sulfonylureas in patients with diabetes due to Kir6.2 mutations. N Engl J Med 2006;355:467–77. 10.1056/NEJMoa061759 16885550

[5] Bowman P, Sulen Åsta, Barbetti F, et al Effectiveness and safety of long-term treatment with sulfonylureas in patients with neonatal diabetes due to KCNJ11 mutations: an international cohort study. Lancet Diabetes Endocrinol 2018;6:637–46. 10.1016/S2213-8587(18)30106-2 29880308 PMC6058077

[6] Lanning MS, Carmody D, Szczerbinski L, et al Hypoglycemia in sulfonylurea-treated KCNJ11-neonatal diabetes: Mild-moderate symptomatic episodes occur infrequently but none involving unconsciousness or seizures. Pediatr Diabetes 2017. 10.1111/pedi.12599 PMC5918230 29205704

[7] Diabetes Genes Sulphonylurea transfer in patients with KCNJ11 and ABCC8 mutations – PNDM. Available: https://www.diabetesgenes.org/about-neonatal-diabetes/su-transfer-in-patients-with-kcnj11-and-abcc8-mutations-pndm/ [Accessed 2 Mar 2019].

[8] Cen J, Sargsyan E, Bergsten P Fatty acids stimulate insulin secretion from human pancreatic islets at fasting glucose concentrations via mitochondria-dependent and -independent mechanisms. Nutr Metab 2016;13 10.1186/s12986-016-0119-5 PMC5006523 27582778

[9] Nolan CJ, Madiraju MSR, Delghingaro-Augusto V, et al Fatty acid signaling in the beta-cell and insulin secretion. Diabetes 2006;55 Suppl 2:S16–23. 10.2337/db06-s003 17130640

[10] Fajans SS, Floyd JC, Knopf RF, et al Effect of amino acids and proteins on insulin secretion in man. Recent Prog Horm Res 1967;23:617–-62. 10.1016/b978-1-4831-9826-2.50017-9 4876487

[11] Zhang T, Li C Mechanisms of amino acid-stimulated insulin secretion in congenital hyperinsulinism. Acta Biochim Biophys Sin 2013;45:36–43. 10.1093/abbs/gms107 23212075 PMC3527006

[12] Gromada J, Ma X, Høy M, et al Atp-Sensitive K+ channel-dependent regulation of glucagon release and electrical activity by glucose in wild-type and SUR1-/- mouse alpha-cells. Diabetes 2004;53 Suppl 3:S181–9. 10.2337/diabetes.53.suppl_3.S181 15561909

[13] Dunn-Meynell AA, Rawson NE, Levin BE Distribution and phenotype of neurons containing the ATP-sensitive K+ channel in rat brain. Brain Res 1998;814:41–54. 10.1016/S0006-8993(98)00956-1 9838037

[14] MacDonald PE, De Marinis YZ, Ramracheya R, et al A K ATP channel-dependent pathway within alpha cells regulates glucagon release from both rodent and human islets of Langerhans. PLoS Biol 2007;5:e143 10.1371/journal.pbio.0050143 17503968 PMC1868042

[15] Gylfe E Glucose control of glucagon secretion: there is more to it than KATP channels. Diabetes 2013;62:1391–3. 10.2337/db13-0193 23613562 PMC3636606

[16] Franklin I, Gromada J, Gjinovci A, et al Beta-Cell secretory products activate alpha-cell ATP-dependent potassium channels to inhibit glucagon release. Diabetes 2005;54:1808–15. 10.2337/diabetes.54.6.1808 15919803

[17] Eisenstein AB, Strack I Amino acid stimulation of glucagon secretion by perifused islets of high-protein-fed rats. Diabetes 1978;27:370–6. 10.2337/diab.27.4.370 346423

[18] Brodovicz KG, Girman CJ, Simonis-Bik AMC, et al Postprandial metabolic responses to mixed versus liquid meal tests in healthy men and men with type 2 diabetes. Diabetes Res Clin Pract 2011;94:449–55. 10.1016/j.diabres.2011.09.002 21955958

[19] Cavallo-Perin P, Aimo G, Mazzillo A, et al Gastric emptying of liquids and solids evaluated by acetaminophen test in diabetic patients with and without autonomic neuropathy. Riv Eur Sci Med Farmacol 1991;13:205–9. 1819847

[20] Fourtner SH, Stanley CA, Kelly A Protein-sensitive hypoglycemia without leucine sensitivity in hyperinsulinism caused by K(ATP) channel mutations. J Pediatr 2006;149:47–52. 10.1016/j.jpeds.2006.02.033 16860127

[21] Li C, Buettger C, Kwagh J, et al A signaling role of glutamine in insulin secretion. J Biol Chem 2004;279:13393–401. 10.1074/jbc.M311502200 14736887

[22] Smart CEM, Evans M, O'Connell SM, et al Both dietary protein and fat increase postprandial glucose excursions in children with type 1 diabetes, and the effect is additive. Diabetes Care 2013;36:3897–902. 10.2337/dc13-1195 24170749 PMC3836096

[23] Cheng-Xue R, Gómez-Ruiz A, Antoine N, et al Tolbutamide controls glucagon release from mouse islets differently than glucose: involvement of K(ATP) channels from both α-cells and δ-cells. Diabetes 2013;62:1612–22. 10.2337/db12-0347 23382449 PMC3636641

[24] Miki T, Liss B, Minami K, et al Atp-Sensitive K+ channels in the hypothalamus are essential for the maintenance of glucose homeostasis. Nat Neurosci 2001;4:507–12. 10.1038/87455 11319559

[25] Evans ML, McCrimmon RJ, Flanagan DE, et al Hypothalamic ATP-sensitive K + channels play a key role in sensing hypoglycemia and triggering counterregulatory epinephrine and glucagon responses. Diabetes 2004;53:2542–51. 10.2337/diabetes.53.10.2542 15448082

[26] Chakera AJ, Hurst PS, Spyer G, et al Molecular reductions in glucokinase activity increase counter-regulatory responses to hypoglycemia in mice and humans with diabetes. Mol Metab 2018;17:17–27. 10.1016/j.molmet.2018.08.001 30146176 PMC6197723

