# Supplementary figures and images for: Patterns of postmeal insulin secretion in individuals with sulfonylurea-treated KCNJ11 neonatal diabetes show predominance of non-KATP-channel pathways
Source: BMJ Open Diabetes Res Care. 2019 Dec 18;7(1):e000721. doi: 10.1136/bmjdrc-2019-000721 (PMC6936449; doi:10.1136/bmjdrc-2019-000721)

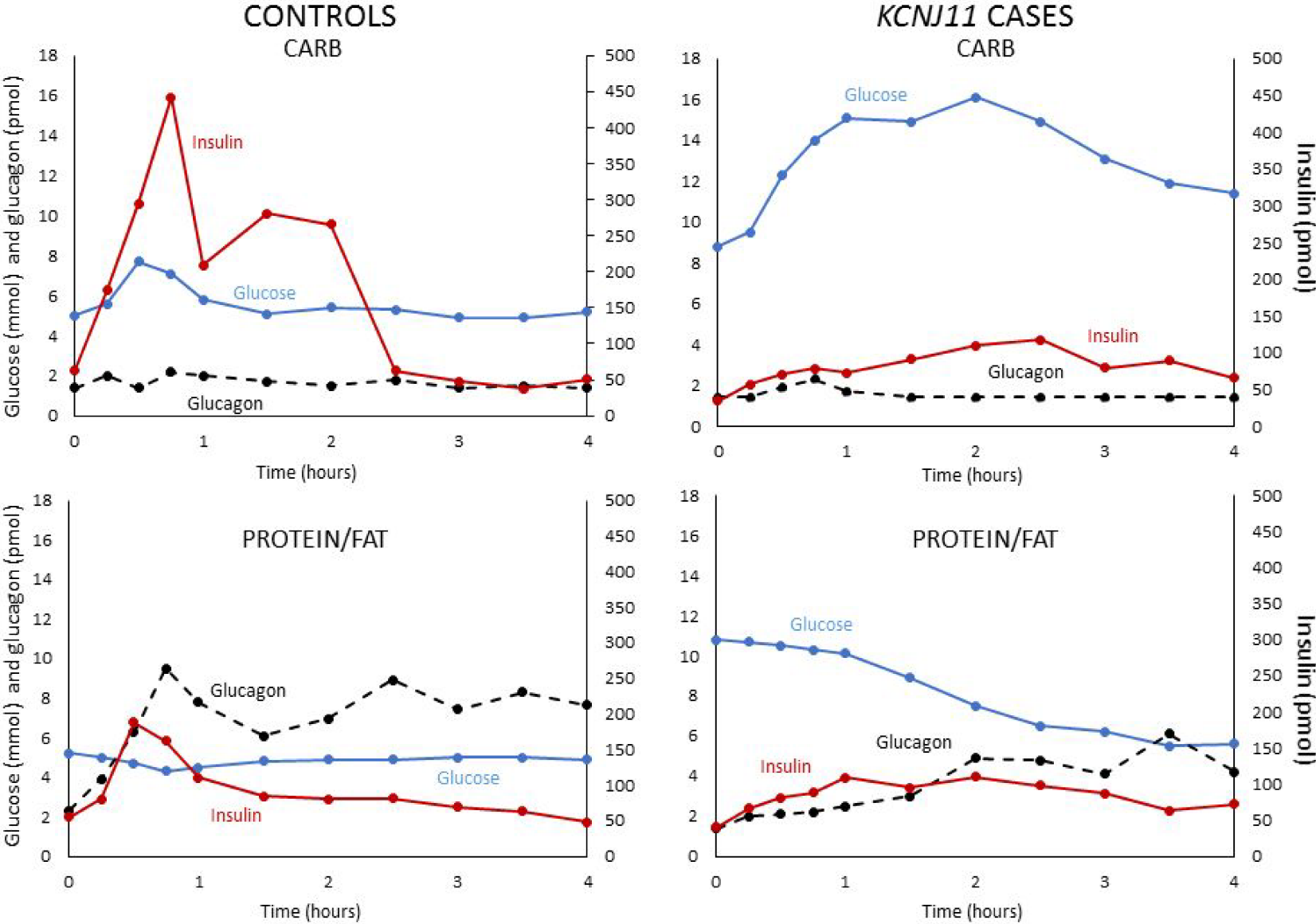

Supplement: Supplementary data [file bmjdrc-2019-000721supp001.pdf]

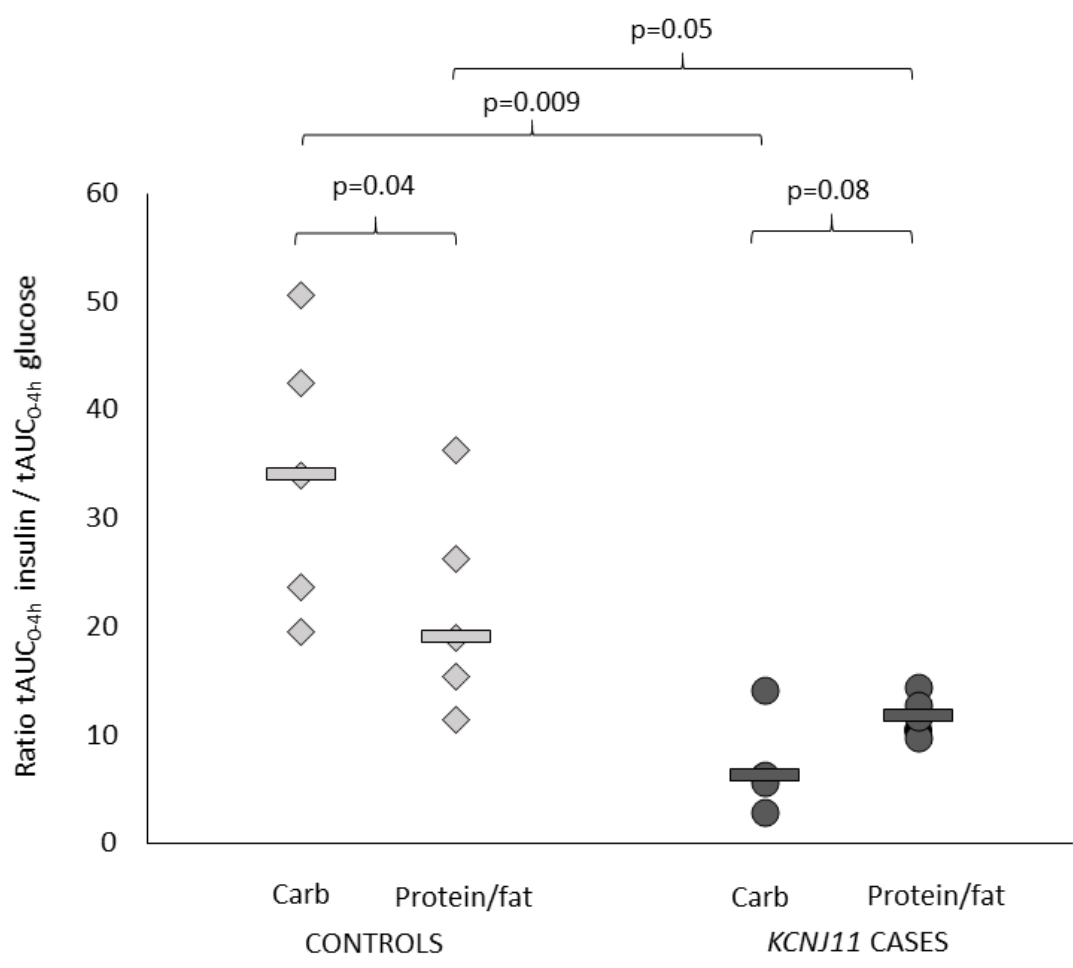

Supplement: Supplementary data [file bmjdrc-2019-000721supp003.pdf]

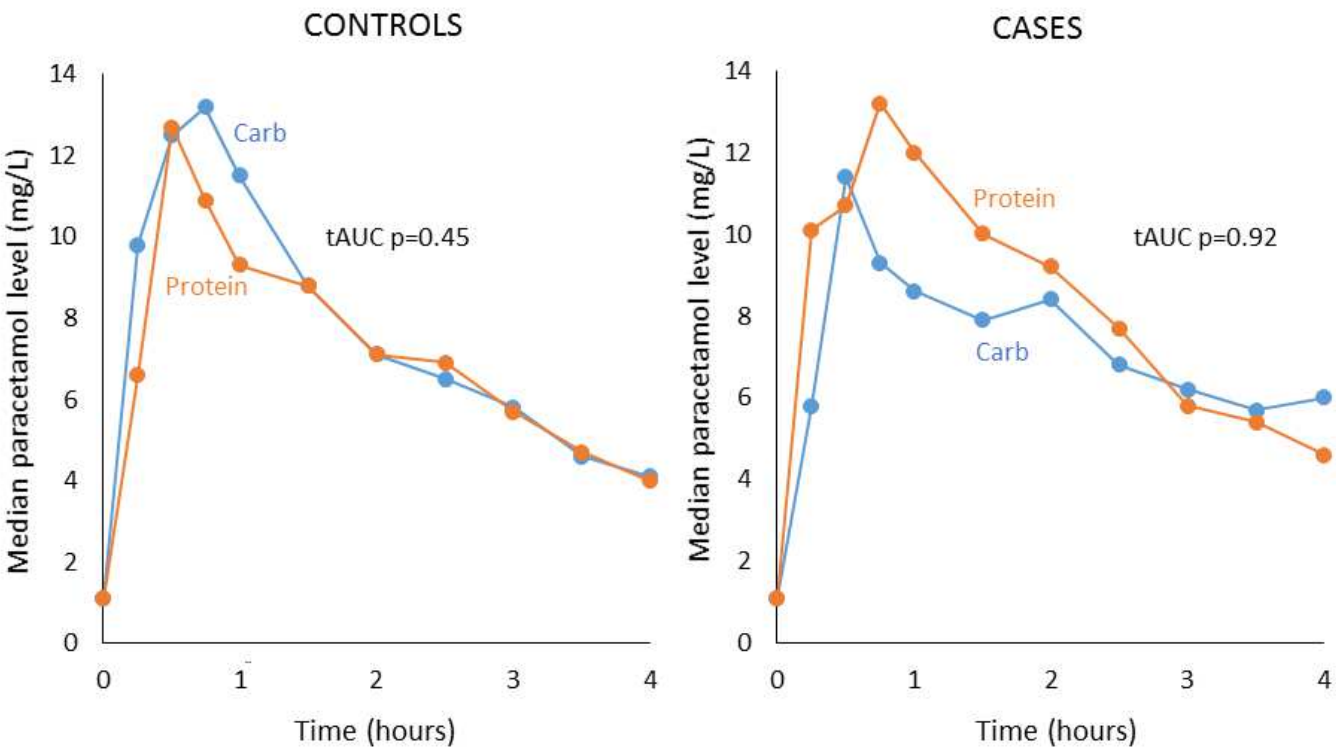

Supplement: Supplementary data [file bmjdrc-2019-000721supp004.pdf]

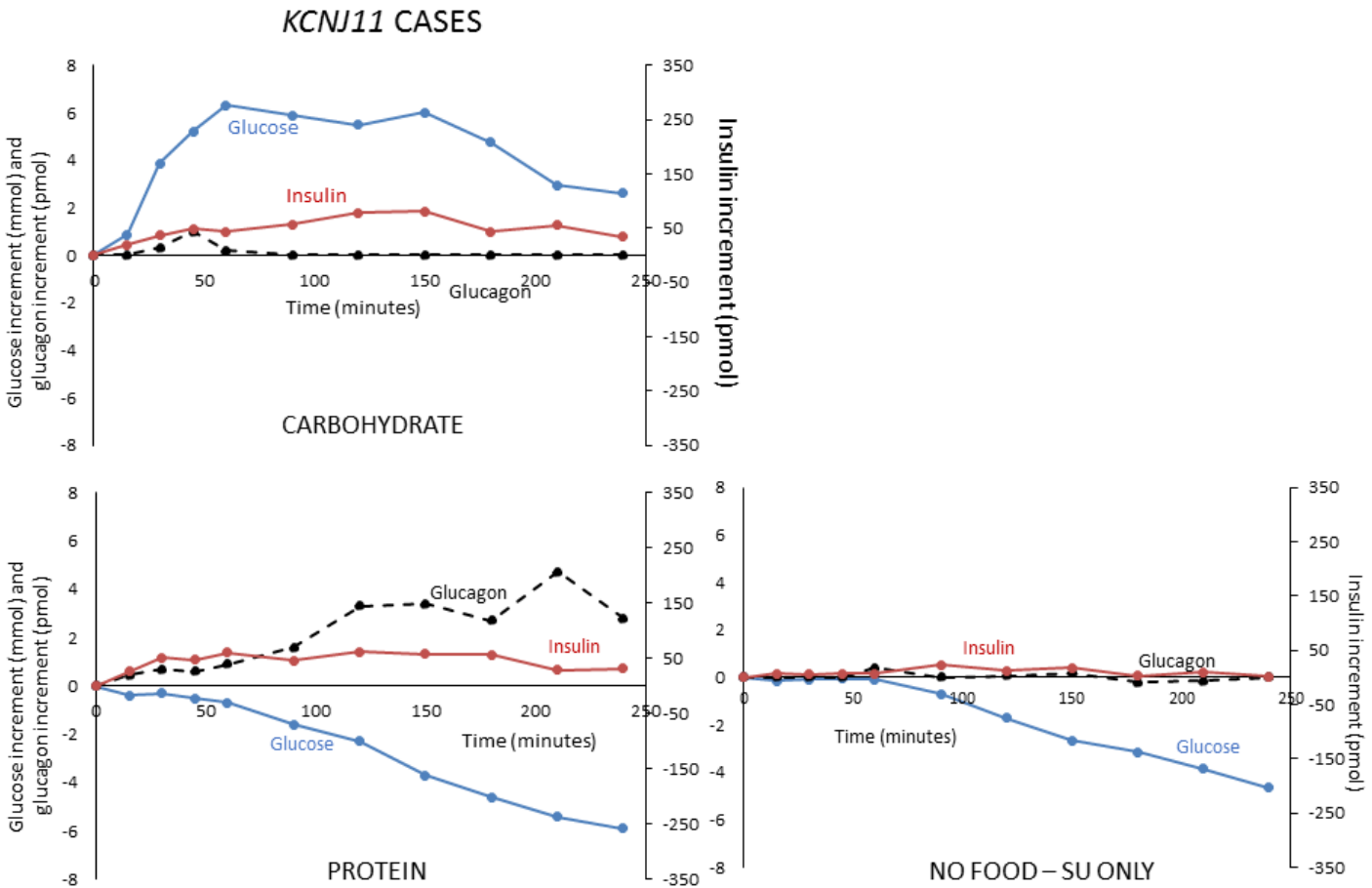

Supplement: Supplementary data [file bmjdrc-2019-000721supp005.pdf]

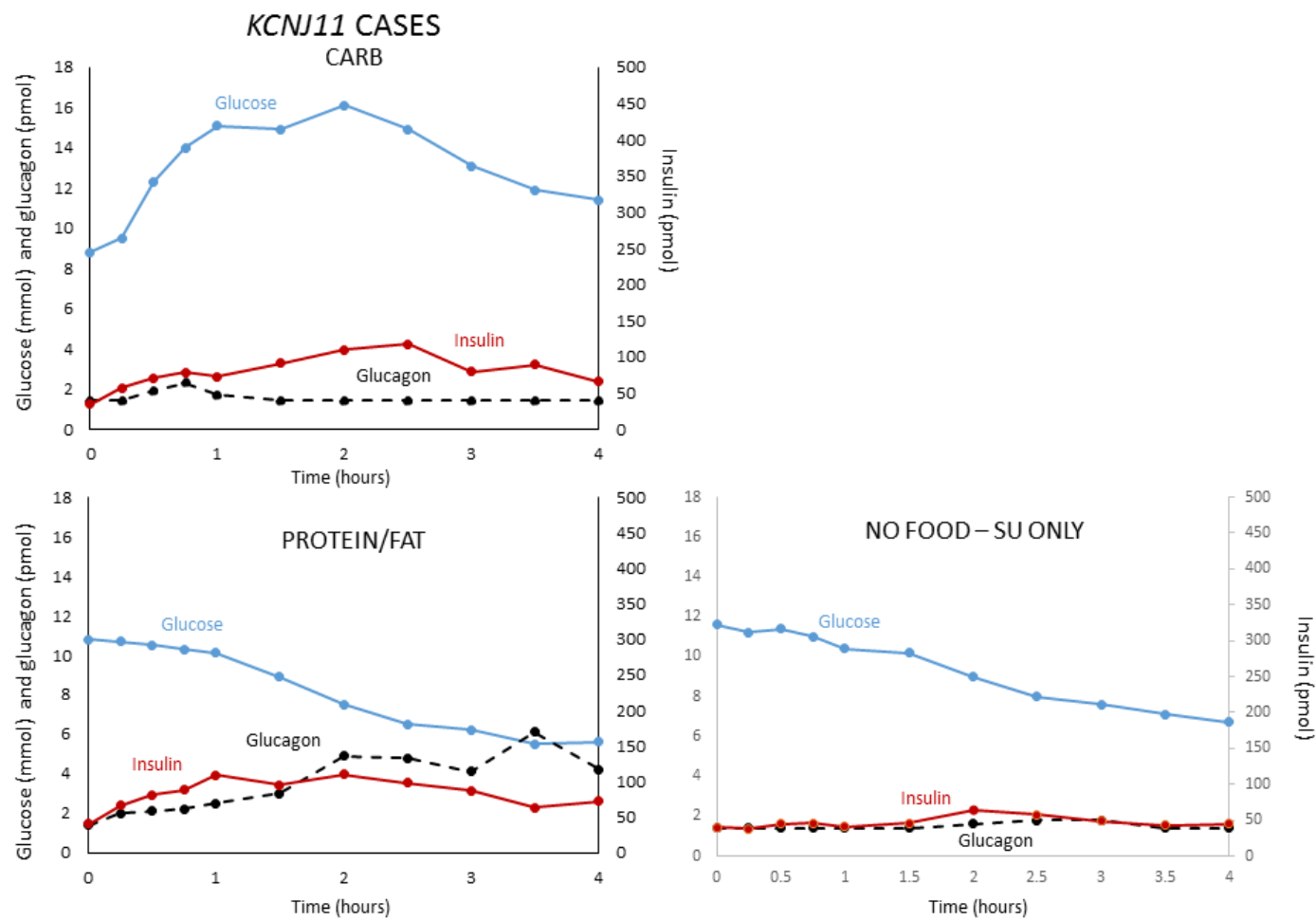

Supplement: Supplementary data [file bmjdrc-2019-000721supp006.pdf]
