# Supplementary material for: Patterns of postmeal insulin secretion in individuals with sulfonylurea-treated KCNJ11 neonatal diabetes show predominance of non-KATP-channel pathways
Source: BMJ Open Diabetes Res Care. 2019 Dec 18;7(1):e000721. doi: 10.1136/bmjdrc-2019-000721 (PMC6936449; doi:10.1136/bmjdrc-2019-000721)
Supplement: Supplementary data [file bmjdrc-2019-000721supp002.pdf]

Supplementary Table 1. Incremental area under the curve (iAUC) for glucose, insulin and glucagon after different meals in *KCNJ11* cases and controls.

**Outcome**	**Group**	**Carb**	**Protein**	**No food**	**P-value carb vs protein**	**P-value protein vs no food**	**P-value carb vs no food**
Glucose iAUC (mmol/L)	Cases	16.7 (8.3-31.6)	-10.9 (-29.1 - -2.2)	-7.6 (-11.8-3.9)	0.04	0.22	0.04
	Controls	1.00 (-0.7-3.3)	-1.15 (-1.5- -0.9)	N/A	0.04	N/A	N/A
Insulin iAUC (pmol/L)	Cases	205 (104-480)	183 (109-316)	39 (13-101)	0.69	0.04	0.04
	Controls	472 (230-992)	70 (8-310)	N/A	0.04	N/A	N/A
Glucagon iAUC (pmol/L)	Cases	0.5 (0.1-1.2)	11.8 (2.2-16.7)	0.2 (-0.2-1.9)	0.04	0.04	0.50
	Controls	0.3 (-2.8-1.7)	18.4 (-6.9-26.3)	N/A	0.14	N/A	N/A
