# Supplementary material for: Patterns of postmeal insulin secretion in individuals with sulfonylurea-treated KCNJ11 neonatal diabetes show predominance of non-KATP-channel pathways
Source: BMJ Open Diabetes Res Care. 2019 Dec 18;7(1):e000721. doi: 10.1136/bmjdrc-2019-000721 (PMC6936449; doi:10.1136/bmjdrc-2019-000721)
Supplement: Supplementary data [file bmjdrc-2019-000721supp007.pdf]

Online only Supplemental Figures - legends

Figure S1 – absolute values insulin, glucagon and glucose in controls without diabetes and *KCNJ11* cases in response to carbohydrate and protein meals. Values shown are medians.

Figure S2 - ratio of total $AUC_{0\text{-}4h}$ insulin / total $AUC_{0\text{-}4h}$ glucose. Controls are shown in grey (diamonds are individuals and lines are group medians). *KCNJ11* cases are shown in black (circles are individuals and lines are group medians).

Figure S3 – paracetamol absorption curves in controls and cases with the carbohydrate (blue) and protein/fat (orange) meals

Figure S4 – incremental glucose, insulin and glucagon in *KCNJ11* cases with sulfonylurea only in the absence of food in comparison to the carbohydrate and protein/fat meal. Values shown are medians.

Figure S5 - absolute glucose insulin, and glucagon and in *KCNJ11* cases with sulfonylurea only in the absence of food in comparison to the carbohydrate and protein/fat meal. Values shown are medians.
